# Secreção não Clássica: Um Possível Mecanismo para Explicar as Elevações da Troponina Cardíaca na Ausência de Infarto Agudo do Miocárdio

**DOI:** 10.36660/abc.20210518

**Published:** 2022-04-07

**Authors:** Jose Manuel Gonzalez-Rayas, Jose Ascencion Hernandez-Hernandez, Rosa del Carmen Lopez-Sanchez, Ana Lilia Rayas-Gomez, Jose Manuel Gonzalez-Yanez

**Affiliations:** 1 School of Medicine and Health Science Monterrey México Tecnologico de Monterrey, School of Medicine and Health Science, Monterrey – México; 2 Hospital San Jose de Queretaro Queretaro México Hospital San Jose de Queretaro, Queretaro – México

**Keywords:** Troponina/uso terapêutico, Ensaios Clínicos Controlados/métodos, Exossomos, Vesiculas Secretórias

*“É importante perceber que, se certas áreas da ciência *

*parecem ser bastante maduras, outras estão em processo de *

*desenvolvimento e outras ainda estão por nascer.”*

***Santiago Ramón y Cajal, ***

***Conselho para um jovem investigador***

## Introdução

Atualmente, os ensaios de troponina cardíaca de alta sensibilidade (hs-cTn) estão disponíveis para uso clínico e fazem parte da definição de infarto agudo do miocárdio (IAM).^1 , 2^ No entanto, a elevação da troponina não se limita ao IAM, já que outras condições relacionadas ao *mismatch* da demanda de oxigênio, dano direto ao miocárdio, aumento de *strain* miocárdico, processos sistêmicos (por ex., sepse), doença neurológica e insuficiência renal também podem resultar em seu aumento.^2^ A troponina agora também pode ser detectada em cenários atípicos, como em exercícios de resistência extenuante, estimulação atrial rápida e ecocardiografia de estresse com dobutamina, bem como em 50% a 100% dos indivíduos saudáveis.^2 , 3^

Do ponto de vista fisiológico, a troponina é um complexo proteico que regula a função miofibrilar, formado por três subunidades: I, T e C. No caso da troponina I e da troponina T, existem 3 isoformas tecido-específicas diferentes: troponina esquelética de contração rápida ( *fast twitch* ), esquelética de contração lenta ( *slow twitch* ) e cardíaca-específica (fsTn, ssTn e cTn).^4^ Por outro lado, a TnC tem duas isoformas, uma presente no músculo esquelético de contração rápida (fsTnC) e outra com expressão tanto no músculo esquelético de contração lenta quanto no músculo cardíaco (ssTnC /cTnC).^4^

Além da necrose celular, vários mecanismos alternativos de liberação têm sido postulados para as isoformas cardíacas da troponina.^3 , 4^ No entanto, nenhum deles explica de forma concisa porque a troponina é liberada em casos não relacionados ao infarto do miocárdio ou se isso é indicativo de dano reversível ou permanente à célula cardíaca. Considerando a atual falta de conhecimento, nosso principal objetivo foi explorar a viabilidade de um novo mecanismo de liberação de troponina cardíaca utilizando uma abordagem de bioinformática.

## Materiais e métodos

### Revisão das evidências existentes

Antes de iniciar a análise, foi realizada uma ampla revisão da literatura em busca de artigos relacionados à secreção de troponina. As bases de dados utilizadas foram PubMed, bioRxiv e OpenGrey. Os artigos foram avaliados primeiramente com base em seu título. Se este fazia alusão à troponina e a um processo secretor, o resumo era lido. Os artigos eram selecionados se o resumo se referisse à troponina e a um processo ou via secretora. Artigos puramente clínicos que não investigaram um mecanismo de liberação da troponina foram excluídos. O processo de triagem e revisão foi realizado por todos os autores. As discordâncias foram resolvidas através de consenso durante reuniões regulares.

### Análise de sequência

Para avaliar a secreção não clássica de troponina, foi utilizado o servidor *SecretomeP 2.0* (http://www.cbs.dtu.dk/services/SecretomeP/).^5^ Essa ferramenta é aplicada para prever se uma determinada proteína sofre secreção sem sinalização de peptídeo. É um método baseado em sequência que, com a ajuda de redes neurais, detecta características específicas comuns a proteínas extracelulares/secretadas.^5^

As sequências canônicas das três subunidades de troponina do músculo esquelético de contração rápida, contração lenta e músculo cardíaco foram obtidas do banco de dados UniProtKB (Arquivo Suplementar 1). As proteínas foram primeiramente examinadas com *SignalP 5.0* , um método comumente utilizado para o reconhecimento de sinalização de peptídeos com base em redes neurais (http://www.cbs.dtu.dk/services/SignalP/).^6^ Esta etapa inicial foi realizada para excluir a via secretora clássica. Em seguida, as sequências foram analisadas com *SecretomeP 2.0* . Finalmente, para avaliar a possibilidade de secreção não clássica do tipo IV (utilizada por proteínas transmembranares que se desviam do Complexo de Golgi), as sequências foram avaliadas utilizando o *TMHMM 2.0* , um programa projetado para detectar hélices transmembranares (http://www.cbs.dtu.dk/services/TMHMM/).^7^ Uma representação gráfica do pipeline de bioinformática que foi seguido é mostrada na Figura Suplementar 1.

## Resultados

### Revisão das evidências existentes

Cerca de 19.900 artigos foram revisados, dos quais 31 foram lidos na íntegra. Após a avaliação do resumo e do texto completo, não foram encontrados artigos relacionados à secreção de troponina. Portanto, que seja de nosso conhecimento, este é o primeiro artigo relatando evidências de secreção não clássica de troponina.

### Análise de sequência

Após analisar as isoformas da troponina, o *SecretomeP 2.0* previu que cTnT, fsTnI, ssTnI, fsTnT e fsTnC eram secretadas de forma não clássica. Nos 5 casos, as proteínas alcançaram um escore de rede neural (escore NN) superior a 0,6, que é o limite mínimo para sequências em mamíferos. Além disso, nenhuma das oito isoformas da troponina mostrou conter uma sinalização de peptídeo ou uma hélice transmembranar, de acordo com *SignalP 5.0* e *TMHMM 2.0* , respectivamente. Os resultados resumidos são apresentados na Tabela 1 . Os resultados completos podem ser encontrados na Tabela Suplementar 1.

**Tabela 1:** – Resultados resumidos obtidos de *SignalP 5.0, SecretomeP 2.0* e *TMHMM 2.0*

Isoforma da Troponina	Identificador de sequências UniProtKB	*SignalP 5.0*	*SecretomeP 2.0*	*TMHMM 2.0*
*Troponina Cardíaca T – cTnT (TNNT2)*	P45379-1	Nenhuma sinalização de peptídeo detectada	Escore NN = **0.746**	Nenhuma hélice transmembranar detectada
*Troponina esquelética de contração rápida I – fsTnI (TNNI2*	P48788-1	Nenhuma sinalização de peptídeo detectada	Escore NN = **0.611**	Nenhuma hélice transmembranar detectada
*Troponina esquelética de contração lenta I – ssTnI (TNNI1)*	P19237-1	Nenhuma sinalização de peptídeo detectada	Escore NN = **0.727**	Nenhuma hélice transmembranar detectada
*Troponina esquelética de contração rápida T – fsTnT (TNNT3)*	P45378-1	Nenhuma sinalização de peptídeo detectada	Escore NN = **0.689**	Nenhuma hélice transmembranar detectada
*Troponina esquelética de contração rápida C – \\\fsTnC (TNNC2)*	P02585-1	Nenhuma sinalização de peptídeo detectada	Escore NN = **0.670**	Nenhuma hélice transmembranar detectada

*As isoformas de troponina com um escore NN acima do limiar de 0,6 para sequências de mamíferos são mostradas. Escore NN: escore de rede neural.*

## Discussão

### Secreção não clássica

A secreção não clássica ou não convencional é uma via de liberação de proteínas. Ao contrário da secreção clássica, ela é independente do retículo endoplasmático (RE)/Complexo de Golgi.^8^ Consequentemente, ela não necessita de uma sinalização de peptídeo, que é uma sequência curta de aminoácidos que conduz a proteína através do processo secretório clássico (mediado pelo RE/Golgi).^9^ Em vez disso, as proteínas secretadas de forma não clássica são liberadas através de uma miríade de mecanismos que podem ser classificados em 4 grupos: tipo I (transporte dependente de poros), tipo II (liberação mediada por transportador ABC), tipo III (liberação através de endossomos/autofagossomos) e tipo IV (desvio do Complexo de Golgi por proteínas transmembranares).^8^ Além disso, outros mecanismos, como exossomos e bolhas, também foram reconhecidos como participantes da secreção não clássica.^9^ Curiosamente, a maioria dos cenários de secreção não-convencional são desencadeados por estresse celular, como inflamação. Alguns exemplos de proteínas que utilizam a via não clássica são IL-1β/IL-1α, FGF-1, FGF-2 e galectinas.^8 , 9^

### Implicações clínicas da secreção não clássica da troponina

**Figura 1:**
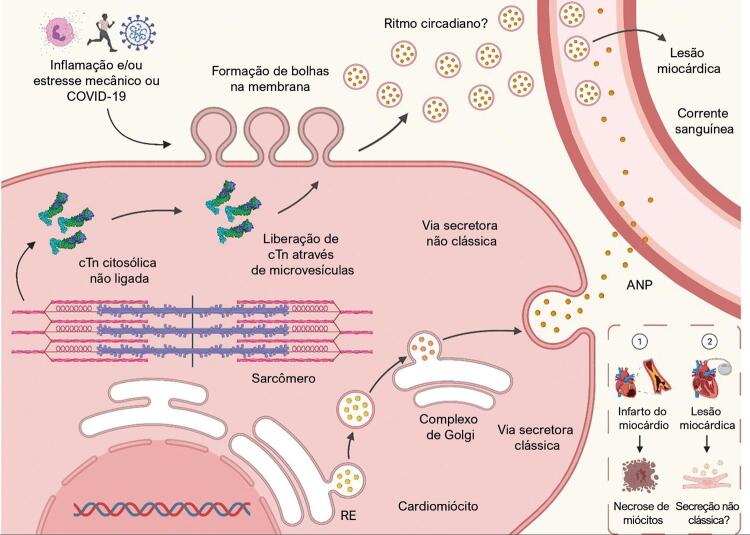
– Ilustração do citosol de um cardiomiócito. A via clássica de secreção é representada pelas vesículas que viajam do retículo endoplasmático para o Complexo de Golgi; as vesículas chegam à membrana plasmática, onde liberam sua carga. A via de secreção não clássica proposta também é mostrada. Inicia-se pela formação de bolhas membranosas como resultado da inflamação e/ou estresse celular que afeta o cardiomiócito. Subsequentemente, a troponina cardíaca citosólica não ligada (cTn) (representando cerca de 2-4% e 6-8% do total de cTnI e cTnT, respectivamente)1 entra nessas bolhas e é liberada como microvesículas. Essas microvesículas entram na corrente sanguínea, onde podem ser detectadas por ensaios de troponina cardíaca de alta sensibilidade. A caixa no canto inferior direito mostra a fisiopatologia de duas condições diferentes em que a troponina se eleva. No primeiro caso, um infarto do miocárdio tipo 1, um evento isquêmico agudo leva à necrose irreversível dos miócitos e à liberação de troponina. No segundo caso, a estimulação atrial rápida induz a liberação de troponina. Se a secreção não clássica participa desse processo permanece uma questão em aberto. Criado com BioRender.com. A estrutura 3D do complexo de troponina foi elaborada com dados do Protein Data Bank ID 1J1E.

Clinicamente, a secreção não clássica de cTnT pode ajudar a resolver o debate em torno dos ensaios de hs-cTn. Uma via de secreção estabelecida para cTn potencialmente explica por que as troponinas são detectadas em indivíduos saudáveis. Além disso, a liberação não clássica de cTn pode contribuir para melhor definir a base patológica da “lesão miocárdica”. Esse termo foi incluído na Quarta definição universal de infarto do miocárdio, sendo um valor de troponina acima do limite superior de referência, a condição sine qua non para seu diagnóstico.^1^ Não é irracional pensar que uma condição de atividade geradora de estresse no célula cardíaca resulta na liberação de troponina através de um processo secretor não clássico ( Figura 1 ). Nesse sentido, as elevações de troponina podem ser o resultado de condições sistêmicas que refletem no coração através de um mecanismo inflamatório ou de estresse celular. Este poderia ser o caso de pacientes com sepse, anemia, câncer, acidente vascular cerebral, convulsões ou após exercício extenuante.^1 , 4^ Curiosamente, a cTnT, mas não a troponina I, mostrou um padrão circadiano de liberação.^2^A liberação de cTnT por células tumorais, possivelmente por vesículas extracelulares, também foi relatada recentemente.^10^

### Limitações da proposta

A principal limitação de nossa proposta é que tanto a troponina I quanto a troponina T cardíaca estão elevadas na lesão miocárdica.^1^ Uma possível explicação poderia ser que a troponina T cardíaca, a subunidade estrutural do complexo da troponina, carrega as outras subunidades na forma de um dímero ou trímero durante o processo secretor não clássico; entretanto, esta é uma suposição hipotética e permanece uma questão em aberto, mas intrigante.

### Limitações do SecretomeP 2.0

O *SecretomeP 2.0* foi criado em 2004 e, após mais de 15 anos, continua a ser um método popular para a avaliação da secreção não clássica.^5^ No entanto, e como acontece com todos os métodos computacionais, os resultados obtidos são preditivos. Dessa forma, eles devem ser interpretados em conjunto com o corpo de evidência experimental existente.

### Evidências que suportam a secreção não clássica de cTn

Vale ressaltar que já existe literatura corroborando nossos achados. A formação de bolhas por cardiomiócitos durante a isquemia, bem como a liberação de troponina através de microvesículas em múltiplas linhagens celulares (na forma de proteína e mRNA) já foram demonstradas anteriormente. As evidências disponíveis estão resumidas na Tabela 2 .

**Tabela 2:** – Evidências experimentais que apoiam a secreção não clássica de troponina cardíaca. A *Vesiclepedia* é um compêndio eletrônico de biomoléculas identificadas em vesículas extracelulares

Evidência Experimental	Referência
Proteína semelhante à troponina secretada por *Meloidogyne incognita* , um nematoide comumente encontrado no solo	Jaubert S, Laffaire JB, Piotte C, Abad P, Rosso M-N, Ledger TN. Direct identification of stylet secreted proteins from root-knot nematodes by a proteomic approach. Molecular and Biochemical Parasitology 121: 205–211, 2002. doi: 10.1016/S0166-6851(02)00034-8
Os cardiomiócitos humanos formam bolhas membranares desencadeadas por anoxia. A liberação reversível de enzimas citosólicas através de bolhas também foi demonstrada.	Hickman PE, Potter JM, Aroney C, Koerbin G, Southcott E, Wu AHB, Roberts MS. Cardiac troponin may be released by ischemia alone, without necrosis. Clinica Chimica Acta 411: 318–323, 2010. doi: 10.1016/j.cca.2009.12.009
Identificação de uma proteína não-caracterizada nos exossomos liberados por cardiomiócitos de ratos sob diferentes estressores (etanol e hipóxia/reoxigenação). A proteína não-caracterizada UniProt ID (E9PTA1) acabou por ser o número de acesso secundário de Tnnc1 (troponina C cardíaca de *Rattus norvegicus* ).	Malik ZA, Kott KS, Poe AJ, Kuo T, Chen L, Ferrara KW, Knowlton AA. Cardiac myocyte exosomes: stability, HSP60, and proteomics. American Journal of Physiology-Heart and Circulatory Physiology 304: H954–H965, 2013. doi: 10.1152/ajpheart.00835.2012
**Evidências da *Vesiclepedia***	
Troponina I tipo 3 (cardíaca) mRNA e proteína do *Homo sapiens* identificada em células de câncer colorretal (microvesículas), células T (exossomos) e urina (vesículas extracelulares)	PubMed IDs: 19930720, 23463506, 25138791
Troponina C tipo 2 (músculo esquelético de contração rápida) Proteína de *Homo sapiens* identificada em células de câncer de ovário (exossomos) e urina (vesículas extracelulares)	PubMed IDs: 24434149, 25138791
Troponina C tipo 2 (músculo esquelético de contração rápida) Proteína de *Mus musculus* identificada em células de melanoma (vesículas extracelulares)	PubMed ID: 29907695
Troponina C tipo 1 (músculo esquelético de contração lenta) Proteína e mRNA de *Homo sapiens* identificados em células de câncer cerebral (vesículas extracelulares), células de câncer colorretal (microvesículas e vesículas extracelulares), células de câncer renal (vesículas extracelulares), células de leucemia (vesículas extracelulares), células de câncer de pulmão (vesículas extracelulares), células de melanoma (vesículas extracelulares) e células de câncer de ovário (vesículas extracelulares)	PubMed IDs: 27894104, 19930720
Troponina I tipo 2 (músculo esquelético de contração rápida) Proteína de *Homo sapiens* identificada na urina (exossomos)	PubMed ID: 22418980
Troponina T tipo 1 (músculo esquelético de contração lenta) mRNA de *Homo sapiens* identificado em células de câncer colorretal (microvesículas) e células de glioblastoma (microvesículas)	PubMed IDs: 19930720, 19011622
Troponina T tipo 3 (músculo esquelético de contração rápida) Proteína de *Homo sapiens* identificada em células de câncer cerebral (vesículas extracelulares), células de câncer de mama (vesículas extracelulares), células de câncer colorretal (vesículas extracelulares), células de câncer renal (vesículas extracelulares), células de melanoma (vesículas extracelulares) e células de câncer de ovário (vesículas extracelulares)	PubMed ID: 27894104

*Mais informações podem ser obtidas aqui: Pathan M, Fonseka P, Chitti SV, et al. Vesiclepedia 2019: a compendium of RNA, proteins, lipids and metabolites in extracellular vesicles. Nucleic Acids Res. 2019;47(D1):D516-D519. doi:10.1093/nar/gky1029.*

### Perspectivas futuras

Pesquisas futuras devem ter como objetivo demonstrar a troponina circulante dentro das microvesículas. Isso pode ser testado em pacientes com infarto agudo do miocárdio (onde a necrose deve ser o principal mecanismo de liberação) versus cenários atípicos apresentando elevação da troponina, como exercício extenuante, sepse e acidente vascular cerebral (onde a secreção não clássica deve conduzir a liberação de troponina). Após o isolamento das microvesículas no plasma, uma possível abordagem para validar a via de secreção não clássica poderia ser o uso de espectrometria de massas seguida por confirmação através de anticorpos específicos.

## Conclusão

Ensaios de alta sensibilidade trouxeram incertezas consideráveis sobre as elevações de cTn. A liberação de troponina devido a causas não relacionadas ao IAM permanece indefinida e seu significado prognóstico exato continua a ser investigado. Como nossa proposta é baseada em evidências *in silico* , ela deve ser confirmada experimentalmente antes que seu significado clínico exato possa ser determinado. Se outras variantes da troponina também entram na via secretora não clássica permanece uma questão em aberto. No entanto, é justo dizer que a secreção não clássica da troponina é uma linha de pesquisa promissora em cardiologia que aguarda ser explorada.

## *Material suplementar

Para informação adicional, por favor,clique aqui


