# Supplementary material for: Secreção não Clássica: Um Possível Mecanismo para Explicar as Elevações da Troponina Cardíaca na Ausência de Infarto Agudo do Miocárdio
Source: Arq Bras Cardiol. 2022 Apr 7;118(4):778–82. [Article in Portuguese] doi: 10.36660/abc.20210518 (PMC9006998; doi:10.36660/abc.20210518)
Supplement: Supplementary file 1 [file 2021-0518_CC_Supplementary-material.pdf]

Supplementary Figure 1.

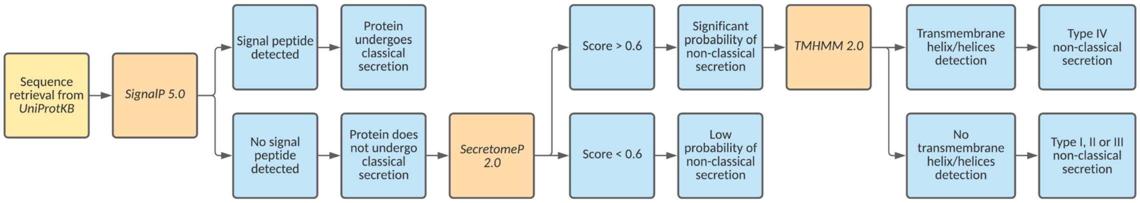

Supplementary Figure 1. Bioinformatic pipeline used to assess the possibility of cardiac troponin non-classical secretion.

Supplementary Table 1

Table 1. Full results obtained from *SignalP 5.0*, *SecretomeP 2.0* and *TMHMM 2.0*.

Troponin isoform	UniProtKB sequence identifier	*SignalP 5.0*	*SecretomeP 2.0*	*TMHMM 2.0*
Cardiac Troponin I - cTnI (TNNI3)	P19429-1	- Signal Peptide (Sec/SPI) = 0.0006<br>- Other = 0.9994	- NN-score = 0.305<br>- Odds = 0.588<br>- WBP = 0.001<br>- Warning = -	- Length = 210<br>- Number of predicted TMHs = 0<br>- Exp number of AAs in TMHs = 0.00091<br>- Exp number, first 60 AAs = 0<br>- Total prob of N-in = 0.14125<br>- Topology = outside 1-210
Cardiac Troponin T - cTnT (TNNT2)	P45379-1	- Signal Peptide (Sec/SPI) = 0.0001<br>- Other = 0.9999	**- NN-score = 0.746**<br>- Odds = 2.723<br>- WBP = 0.005<br>- Warning = -	- Length = 298<br>- Number of predicted TMHs = 0<br>- Exp number of AAs in TMHs = 0<br>- Exp number, first 60 AAs = 0<br>- Total prob of N-in = 0.02083<br>- Topology = outside 1-298
Slow-twitch skeletal and cardiac Troponin C - ssTnC/cTnC (TNNC1)	P63316-1	- Signal Peptide (Sec/SPI) = 0.0004<br>- Other = 0.9996	- NN-score = 0.318<br>- Odds = 0.588<br>- WBP = 0.001<br>- Warning = -	- Length = 161<br>- Number of predicted TMHs = 0<br>- Exp number of AAs in TMHs = 0.00181<br>- Exp number, first 60 AAs = 0.00181<br>- Total prob of N-in = 0.23980<br>- Topology = outside 1-161
Fast-twitch skeletal Troponin I - fsTnI (TNNI2)	P48788-1	- Signal Peptide (Sec/SPI) = 0.0013<br>- Other = 0.9987	**- NN-score = 0.611**<br>- Odds = 1.590<br>- WBP = 0.003<br>- Warning = -	- Length = 182<br>- Number of predicted TMHs = 0<br>- Exp number of AAs in TMHs = 0.00018<br>- Exp number, first 60 AAs = 0<br>- Total prob of N-in = 0.35917<br>- Topology = outside 1-182
Slow-twitch skeletal Troponin I - ssTnI (TNNI1)	P19237-1	- Signal Peptide (Sec/SPI) = 0.0017<br>- Other = 0.9983	**- NN-score = 0.727**<br>- Odds = 2.465<br>- WBP = 0.005<br>- Warning = -	- Length = 187<br>- Number of predicted TMHs = 0<br>- Exp number of AAs in TMHs = 0.00017<br>- Exp number, first 60 AAs = 0<br>- Total prob of N-in = 0.11905<br>- Topology = outside 1-187
Fast-twitch	P45378-1	- Signal Peptide	**- NN-score = 0.689**	- Length = 269

skeletal Troponin T - fsTnT (TNNT3)		(Sec/SPI) = 0.0001<br>- Other = 0.9999	- Odds = 2.188<br>- WBP = 0.004<br>- Warning = -	- Number of predicted TMHs = 0<br>- Exp number of AAs in TMHs = 0<br>- Exp number, first 60 AAs = 0<br>- Total prob of N-in = 0.03713<br>- Topology = outside 1-269
Slow-twitch skeletal Troponin T - ssTnT (TNNT1)	P13805-1	- Signal Peptide (Sec/SPI) = 0.0003<br>- Other = 0.9997	- NN-score = 0.450<br>- Odds = 0.951<br>- WBP = 0.002<br>- Warning = -	- Length = 278<br>- Number of predicted TMHs = 0<br>- Exp number of AAs in TMHs = 0.00044<br>- Exp number, first 60 AAs = 0<br>- Total prob of N-in = 0.06059<br>- Topology = outside 1-278
Fast-twitch skeletal Troponin C - fsTnC (TNNC2)	P02585-1	- Signal Peptide (Sec/SPI) = 0.0006<br>- Other = 0.9994	**- NN-score = 0.670**<br>- Odds = 1.988<br>- WBP = 0.004<br>- Warning = -	- Length = 160<br>- Number of predicted TMHs = 0<br>- Exp number of AAs in TMHs = 0<br>- Exp number, first 60 AAs = 0<br>- Total prob of N-in = 0.40544<br>- Topology = outside 1-160

Full results obtained from *SignalP 5.0*, *SecretomeP 2.0* and *TMHMM 2.0*. Isoforms with a NN-score above the 0.6 threshold are shown in italics and bold. Sec/SPI= probability of a Sec signal peptide within the protein, Other= globular protein with no signal peptide or transmembrane protein, NN-score= neural network score, WBP= Weighted by prior, Warning= reports the presence of a signal peptide (if any) within the sequence, TMHs= transmembrane helices, Exp= expected, AAs= amino acids, Exp number, first 60 AAs= number of amino acids within a predicted TMH that locate among the first 60 AAs of the sequence, Total prob of N-in= likelihood that the amino-terminus of the protein faces the cytoplasmic part of the membrane, Topology= predicted TMHs position along the AA sequence. In this case, since no THMs were found, all the proteins were cataloged as being *outside* from the start amino acid to the end one.
