# Supplementary material for: Secreção não Clássica: Um Possível Mecanismo para Explicar as Elevações da Troponina Cardíaca na Ausência de Infarto Agudo do Miocárdio
Source: Arq Bras Cardiol. 2022 Apr 7;118(4):778–82. [Article in Portuguese] doi: 10.36660/abc.20210518 (PMC9006998; doi:10.36660/abc.20210518)
Supplement: Translation

# Translation: Non-Classical Secretion: A Possible Mechanism to Explain Cardiac Troponin Elevations in the Absence of Acute Myocardial Infarction

**Authors:** Jose Manuel Gonzalez-Rayas, Jose Ascencion Hernandez-Hernandez, Rosa del Carmen Lopez-Sanchez, Ana Lilia Rayas-Gomez, Jose Manuel Gonzalez-Yanez

**Affiliations:** 1 Mexico Tecnologico de Monterrey, School of Medicine and Health Science, Monterrey – Mexico; 2 Mexico Hospital San Jose de Queretaro, Queretaro – Mexico

**Keywords:** Troponin, non-classical secretion, high-sensitivity cardiac troponin assays, exosomes, microvesicles

*“It is important to realize that if certain areas of science*

*appear to be quite mature, others are in the process of*

*development, and yet others remain to be born.”*

***Santiago Ramón y Cajal,***

***Advice for a young investigator***

## Introduction

Nowadays, high-sensitivity cardiac troponin (hs-cTn) assays are available for clinical use and are part of the definition of acute myocardial infarction (AMI).^1 , 2^However, troponin elevation is not limited to AMI, since other conditions related to oxygen demand mismatch, direct myocardial damage, increased myocardial strain, systemic processes (i.e. sepsis), neurological disease and renal failure can also result in its increase.^2^Troponin can now also be detected in atypical scenarios, such as in strenuous endurance exercise, rapid atrial pacing, and dobutamine stress echocardiography, as well as in 50% to 100% of healthy subjects.^2 , 3^

From a physiological point of view, troponin is a protein complex that regulates myofibrillar function, consisting of three subunits: I, T and C. In the case of troponin I and troponin T, there are three different tissue-specific isoforms: fast-twitch skeletal, slow-twitch skeletal and cardiac specific (fsTn, ssTn and cTn).^4^Conversely, TnC has two isoforms, one present in fast-twitch skeletal muscle (fsTnC) and one that expresses in both slow-twitch skeletal and cardiac muscle (ssTnC/cTnC).^4^

Apart from cell necrosis, several alternative release mechanisms have been postulated for the cardiac isoforms of troponin.^3 , 4^However, none of them concisely explains why troponin is released in cases unrelated to myocardial infarction or whether this is indicative of reversible or permanent damage to the cardiac cell. In light of the current lack of knowledge, our main objective was to explore the feasibility of a novel mechanism for cardiac troponin release using a bioinformatical approach.

## Materials and Methods

### Review of the existing evidence

Before starting the analysis, a comprehensive review of the literature was conducted in search of articles related to troponin secretion. The searched databases were PubMed, bioRxiv and OpenGrey. Articles were first assessed based on their title. If it mentioned troponin and a secretory process, the abstract was read. Articles were selected if the abstract mentioned troponin and a secretory process or pathway. Purely clinical articles that did not investigate a mechanism of troponin release were excluded. The screening and review processes were performed by all authors. Disagreements were solved by consensus during regular meetings.

### Sequence analysis

To assess for non-classical secretion of troponin, the *SecretomeP 2.0* server was used (http://www.cbs.dtu.dk/services/SecretomeP/).^5^This tool is applied to predict whether a certain protein undergoes secretion without a signal peptide. It is a sequence-based method that, with the help of neural networks, detects specific features common to extracellular/secreted proteins.^5^

The canonical sequences from the three subunits of troponin from fast-skeletal, slow-skeletal and cardiac muscle were retrieved from the UniProtKB database (Supplementary File 1). The proteins were first assessed with *SignalP 5.0* , a commonly used method for the recognition of signal peptides based on neural networks (http://www.cbs.dtu.dk/services/SignalP/).^6^This initial step was performed to rule out the classical secretory pathway. Afterwards, the sequences were analyzed with *SecretomeP 2.0* . Finally, to assess the possibility of type IV non-classical secretion (used by transmembrane proteins that bypass the Golgi apparatus), the sequences were evaluated using *TMHMM 2.0* , a program designed to detect transmembrane helices (http://www.cbs.dtu.dk/services/TMHMM/).^7^A graphical representation of the followed bioinformatic pipeline can be found in Supplementary Figure 1 .

**Figure 1:** – Illustration of the cytosol of a cardiomyocyte. The classical pathway of secretion is depicted by the vesicles traveling from the endoplasmic reticulum to the Golgi apparatus; the vesicles arrive at the plasma membrane, where they release their load. The proposed non-classical pathway of secretion is also shown. It starts with the formation of membranous blebs as a result of inflammation and/or cell stress, affecting the cardiomyocyte. Subsequently, unbound cytosolic cardiac troponin (cTn) (representing around 2-4% and 6-8% of the total cTnI and cTnT, respectively)1 enters these blebs and is released as microvesicles. These microvesicles enter the bloodstream, where they can be detected by high-sensitivity cardiac troponin assays. The box on the lower right corner shows the physiopathology of two different conditions where troponin increases. In the first case, a type 1 myocardial infarction case, an acute ischemic event leads to irreversible myocyte necrosis and troponin release. In the second case, rapid atrial pacing induces troponin release. Whether non-classical secretion participates in this process remains an open question. Created with BioRender.com. The 3D structure of the troponin complex was created with data from Protein Data Bank ID 1J1E.

## Results

### Review of the existing evidence

Nearly 19,900 articles were reviewed, of which 31 were read in full. After abstract and full text assessment, no articles were found to be related to troponin secretion. Hence, to the best of our knowledge, this is the first paper reporting evidence of troponin non-classical secretion.

### Sequence analysis

After analyzing the troponin isoforms, *SecretomeP 2.0* predicted cTnT, fsTnI, ssTnI, fsTnT and fsTnC to be non-classically secreted. In the 5 cases, the proteins achieved a neural network score (NN-score) greater than 0.6, which is the minimum threshold for mammalian sequences. Additionally, none of the eight troponin isoforms were found to contain a signal peptide or a transmembrane helix, according to *SignalP 5.0* and *TMHMM 2.0* , respectively. The summarized results are shown in Table 1 . The complete results can be found in Supplementary Table 1 .

**Table 1:** – Summarized results obtained with *SignalP 5.0, SecretomeP 2.0* and *TMHMM 2.0*

Troponin isoform	UniProtKB sequence identifier	*SignalP 5.0*	*SecretomeP 2.0*	*TMHMM 2.0*
*Cardiac Troponin T – cTnT (TNNT2)*	P45379-1	No signal peptide detected	**NN-score = 0.746**	No transmembrane helices detected
*Fast-twitch skeletal Troponin I – fsTnI (TNNI2)*	P48788-1	No signal peptide detected	**NN-score = 0.611**	No transmembrane helices detected
*Slow-twitch skeletal Troponin I – ssTnI (TNNI1)*	P19237-1	No signal peptide detected	**NN-score = 0.727**	No transmembrane helices detected
*Fast-twitch skeletal Troponin T – fsTnT (TNNT3)*	P45378-1	No signal peptide detected	**NN-score = 0.689**	No transmembrane helices detected
*Fast-twitch skeletal Troponin C – fsTnC (TNNC2)*	P02585-1	No signal peptide detected	**NN-score = 0.670**	No transmembrane helices detected

*The troponin isoforms with a NN-score above the 0.6 threshold for mammalian sequences are shown. NN-score: neural network score.*

## Discussion

### Non-classical secretion

Non-classical or unconventional secretion is a pathway of protein release. Contrary to classical secretion, it is independent of the Endoplasmic Reticulum (ER)/Golgi apparatus.^8^Consequently, it does not require a signal peptide, which is a short sequence of amino acids that leads the protein through the classical (ER/Golgi-mediated) secretory process.^9^Instead, non-classically secreted proteins are released through a myriad of mechanisms that can be classified into 4 groups: type I (pore-dependent transport), type II (ABC transporter-mediated release), type III (released from endosomes/autophagosomes) and type IV (Golgi bypass by transmembrane proteins).^8^Moreover, other mechanisms, such as exosomes and blebs, have also been recognized to take part in non-classical secretion.^9^Interestingly, most of the scenarios of unconventional secretion are triggered by cell stress, such as inflammation. Some examples of proteins that use the non-classical pathway are IL-1β/IL-1α, FGF-1, FGF-2, and galectins.^8 , 9^

### Clinical implications of troponin non-classical secretion

Clinically, cTnT non-classical secretion could help to solve the debate around hs-cTn assays. An established secretion pathway for cTn potentially explains why troponins are detected in healthy subjects. In addition, cTn non-classical release might contribute to better define the pathological basis of “myocardial injury”. This term was included in the fourth universal definition of myocardial infarction, with a troponin value above the upper reference limit being the required condition for its diagnosis.^1^It is not unreasonable to think that an activity condition that generates stress in the cardiac cell results in the release of troponin through a non-classical secretory process ( Figure 1 ). In this regard, troponin elevations might be the result of systemic conditions reflecting on the heart through an inflammatory or cell stress mechanism. This could be the case of patients with sepsis, anemia, cancer, stroke, seizures, or after strenuous exercise.^1 , 4^Interestingly, cTnT, but not I, has shown a circadian pattern of release.^2^cTnT release by tumor cells, possibly by extracellular vesicles, has also been recently reported.^10^

### Proposal limitations

The main limitation of our proposal is that both cardiac troponin I and troponin T are elevated in myocardial injury.^1^A possible explanation could be that cardiac troponin T, the structural subunit of the troponin complex, carries the other subunits in the form of a dimer or trimer along the non-classical secretory process; however, this is a hypothetical assumption and remains an open but intriguing question.

### SecretomeP 2.0 limitations

*SecretomeP 2.0* was created in 2004, and after more than 15 years it remains a popular method for the assessment of non-classical secretion.^5^However, and as it happens with all computational methods, the results obtained are predictions. Thus, they should be interpreted together with the existing experimental body of evidence.

### Evidence supporting cTn non-classical secretion

It is worth mentioning that there is some literature supporting our findings. Bleb formation by cardiomyocytes during ischemia, as well as troponin release through microvesicles in multiple cell lines (in both protein and mRNA form) have been previously demonstrated. Available evidence is summarized in Table 2 .

**Table 2:** – Experimental evidence supporting cardiac troponin non-classical secretion. *Vesiclepedia* is an electronic compendium of biomolecules identified in extracellular vesicles

Experimental evidence	Reference
Troponin-like protein secreted by Meloidogyne incognita, a root-knot nematode	Jaubert S, Laffaire JB, Piotte C, Abad P, Rosso M-N, Ledger TN. Direct identification of stylet secreted proteins from root-knot nematodes by a proteomic approach. Molecular and Biochemical Parasitology 121: 205–211, 2002. doi: 10.1016/S0166-6851(02)00034-8
Human cardiomyocytes form membranous blebs triggered by anoxia. Reversible cytosolic enzyme release by means of blebs has also been shown.	Hickman PE, Potter JM, Aroney C, Koerbin G, Southcott E, Wu AHB, Roberts MS. Cardiac troponin may be released by ischemia alone, without necrosis. Clinica Chimica Acta 411: 318–323, 2010. doi: 10.1016/j.cca.2009.12.009
Identification of an uncharacterized protein in the exosomes released by rat cardiomyocytes under different stressors (ethanol and hypoxia/reoxygenation). The uncharacterized protein UniProt ID (E9PTA1) turned out to be the secondary accession number of Tnnc1 (cardiac troponin C of Rattus norvegicus).	Malik ZA, Kott KS, Poe AJ, Kuo T, Chen L, Ferrara KW, Knowlton AA. Cardiac myocyte exosomes: stability, HSP60, and proteomics. American Journal of Physiology-Heart and Circulatory Physiology 304: H954–H965, 2013. doi: 10.1152/ajpheart.00835.2012
**Evidence from Vesiclepedia**	
Troponin I type 3 (cardiac) Homo sapiens mRNA and protein identified in colorectal cancer cells (microvesicles), T cells (exosomes) and urine (extracellular vesicles)	PubMed IDs: 19930720, 23463506, 25138791
Troponin C type 2 (fast-twitch skeletal muscle) Homo sapiens protein identified in ovarian cancer cells (exosomes) and urine (extracellular vesicles)	PubMed IDs: 24434149, 25138791
Troponin C type 2 (fast-twitch skeletal muscle) Mus musculus protein identified in melanoma cells (extracellular vesicles)	PubMed ID: 29907695
Troponin C type 1 (slow-twitch skeletal muscle) Homo sapiens protein and mRNA identified in brain cancer cells (extracellular vesicles), colorectal cancer cells (microvesicles and extracellular vesicles), kidney cancer cells (extracellular vesicles), leukemia cells (extracellular vesicles), lung cancer cells (extracellular vesicles), melanoma cells (extracellular vesicles) and ovarian cancer cells (extracellular vesicles)	PubMed IDs: 27894104, 19930720
Troponin I type 2 (fast-twitch skeletal muscle) Homo sapiens protein identified in urine (exosomes)	PubMed ID: 22418980
Troponin T type 1 (slow-twitch skeletal muscle) Homo sapiens mRNA identified in colorectal cancer cells (microvesicles) and glioblastoma cells (microvesicles)	PubMed IDs: 19930720, 19011622
Troponin T type 3 (fast-twitch skeletal muscle) Homo sapiens protein identified in brain cancer cells (extracellular vesicles), breast cancer cells (extracellular vesicles), colorectal cancer cells (extracellular vesicles), kidney cancer cells (extracellular vesicles), melanoma cells (extracellular vesicles) and ovarian cancer cells (extracellular vesicles)	PubMed ID: 27894104

*More information can be found here: Pathan M, Fonseka P, Chitti SV, et al. Vesiclepedia 2019: a compendium of RNA, proteins, lipids and metabolites in extracellular vesicles. Nucleic Acids Res. 2019;47(D1):D516-D519. doi:10.1093/nar/gky1029.*

### Future perspectives

Further research should aim at demonstrating circulating troponin within microvesicles. This could be tested in patients with acute myocardial infarction (where necrosis should be the leading release mechanism) versus atypical scenarios showing troponin elevation such as strenuous exercise, sepsis and stroke (where non-classical secretion should drive troponin release). After the isolation of plasma microvesicles, a possible approach to validate the non-classical secretion pathway could be the use of mass spectrometry followed by confirmation through specific antibodies.

## Conclusion

High-sensitivity assays have brought considerable uncertainty about cTn elevations. Troponin liberation due to non-AMI related causes remains elusive and its exact prognostic significance continues to be investigated. Since our proposal is based on *in silico* evidence, it should be experimentally confirmed before its exact clinical significance can be ascertained. Whether other troponin variants also enter the non-classical secretory pathway is also an open question. Nonetheless, it is fair to say that troponin non-classical secretion is a promising research field in cardiology that awaits to be explored.

## *Supplemental Materials

For additional information, please click here.
